# Beneficial effects of long-term intravenous iron therapy with ferric carboxymaltose in patients with symptomatic heart failure and iron deficiency^†^

**DOI:** 10.1093/eurheartj/ehu385

**Published:** 2014-08-31

**Authors:** Piotr Ponikowski, Dirk J. van Veldhuisen, Josep Comin-Colet, Georg Ertl, Michel Komajda, Viacheslav Mareev, Theresa McDonagh, Alexander Parkhomenko, Luigi Tavazzi, Victoria Levesque, Claudio Mori, Bernard Roubert, Gerasimos Filippatos, Frank Ruschitzka, Stefan D. Anker

**Affiliations:** 1 Department of Heart Diseases, Medical University, Wroclaw, Poland; 2 Department of Cardiology, Center for Heart Diseases, Clinical Military Hospital, Weigla 5 53-114, Wroclaw, Poland; 3 Department of Cardiology, University Medical Center Groningen, University of Groningen, Groningen, The Netherlands; 4 Heart Diseases Biomedical Research Group, IMIM (Hospital del Mar Medical Research Institute), Barcelona, Spain; 5 Department of Internal Medicine I, University Hospital Würzburg, Würzburg, Germany; 6 Comprehensive Heart Failure Center, University of Würzburg, Würzburg, Germany; 7 CHU Pitié-Salpêtrière, Institut de Cardiologie, Paris, France; 8 Lomonosov Moscow State University, Moscow, Russia; 9 Department of Cardiology, King's College Hospital, Denmark Hill, London SE5 9RS, UK; 10 Ukranian Strazhesko Institute of Cardiology, 5, Narodnoko Opolchenia St, Kiev 03151, Ukraine; 11 Maria Cecilia Hospital, GVM Care&Research—E.S. Health Science Foundation, Cotignola, Italy; 12 Vifor Pharma, Glattbrugg, Switzerland; 13 Athens University Hospital Attikon, Athens, Greece; 14 Department of Cardiology, University Hospital Zurich, Switzerland; 15 Department of Innovative Clinical Trials, University Medical Centre Göttingen, Göttingen, Germany

**Keywords:** Heart failure, Iron deficiency, Ferric carboxymaltose

## Abstract

**Aim:**

The aim of this study was to evaluate the benefits and safety of long-term i.v. iron therapy in iron-deficient patients with heart failure (HF).

**Methods and results:**

CONFIRM-HF was a multi-centre, double-blind, placebo-controlled trial that enrolled 304 ambulatory symptomatic HF patients with left ventricular ejection fraction ≤45%, elevated natriuretic peptides, and iron deficiency (ferritin <100 ng/mL or 100–300 ng/mL if transferrin saturation <20%). Patients were randomized 1 : 1 to treatment with i.v. iron, as ferric carboxymaltose (FCM, *n* = 152) or placebo (saline, *n* = 152) for 52 weeks. The primary end-point was the change in 6-min-walk-test (6MWT) distance from baseline to Week 24. Secondary end-points included changes in New York Heart Association (NYHA) class, Patient Global Assessment (PGA), 6MWT distance, health-related quality of life (QoL), Fatigue Score at Weeks 6, 12, 24, 36, and 52 and the effect of FCM on the rate of hospitalization for worsening HF. Treatment with FCM significantly prolonged 6MWT distance at Week 24 (difference FCM vs. placebo: 33 ± 11 m, *P* = 0.002). The treatment effect of FCM was consistent in all subgroups and was sustained to Week 52 (difference FCM vs. placebo: 36 ± 11 m, *P* < 0.001). Throughout the study, an improvement in NYHA class, PGA, QoL, and Fatigue Score in patients treated with FCM was detected with statistical significance observed from Week 24 onwards. Treatment with FCM was associated with a significant reduction in the risk of hospitalizations for worsening HF [hazard ratio (95% confidence interval): 0.39 (0.19–0.82), *P* = 0.009]. The number of deaths (FCM: 12, placebo: 14 deaths) and the incidence of adverse events were comparable between both groups.

**Conclusion:**

Treatment of symptomatic, iron-deficient HF patients with FCM over a 1-year period resulted in sustainable improvement in functional capacity, symptoms, and QoL and may be associated with risk reduction of hospitalization for worsening HF (ClinicalTrials.gov number NCT01453608).

**See page 645 for the editorial comment on this article (doi:10.1093/eurheartj/ehu392)**

## Introduction

With the constantly increasing prevalence and incidence, heart failure (HF) has now become an epidemic problem carrying relevant medical, social and economic consequences.^1^ Despite recent developments in HF management, the morbidity and mortality in this clinical syndrome remain unacceptably high and many patients suffer from debilitating symptoms adversely affecting their quality of life.^2–4^ Cardiovascular and non-cardiovascular co-morbidities often complicate the natural course of HF with deleterious impact on clinical status, symptoms, and HF progression, thus constituting targets for potential intervention.^2,5^

Iron deficiency (ID) is one of the most common nutritional deficiencies worldwide, affecting one-third of the general population.^6^ Several chronic disorders may be complicated by ID,^6–9^ but only recently ID has been also reported as a frequent co-morbidity in stable HF patients regardless of ejection fraction^10,11,12^ and in patients admitted to hospital due to worsening HF.^13^ Mechanisms underlying the development of ID in HF have not been rigorously investigated, but ID may be a consequence of impaired iron absorption, augmented gastro-intestinal loss, and reduced availability of utilizable iron from the reticuloendothelial system.^14^ Heart failure complicated with ID is associated with impaired functional capacity, poor quality of life, and increased mortality.^10,11,13–15^ Interestingly, opposite to the traditional view, deleterious consequences of ID in HF syndrome are irrespective of anaemia and other important confounders (e.g. age, severity of the disease, renal function).^10–15^ Thus, correction of ID itself can be considered an attractive therapeutic target in HF, and this hypothesis has been recently tested in a few clinical studies.^14,16^ These trials, however, suffer from several drawbacks: the vast majority was single-centre studies with heterogeneous design (open-label vs. placebo-controlled, treating only patients with anaemia vs. all ID patients, typically with short-duration of therapy). While i.v. iron therapy in iron-deficient stable HF patients^14,16^ appears beneficial, there are still uncertainties on the appropriate use of iron in HF. In particular, these uncertainties include longer-term sustainability of beneficial effects, safety, and potential impact on outcomes. Thus, we designed the CONFIRM-HF (Ferric CarboxymaltOse evaluatioN on perFormance in patients with IRon deficiency in coMbination with chronic Heart Failure) to address these questions.

## Methods

### Study design and oversight

Between September 2011 and February 2013, 304 eligible patients were enrolled from 41 sites in 9 countries. The study design has been published.^17^ The protocol was approved by the institutional review board at each participating centre and conducted in accordance with the principles of the Declaration of Helsinki (1996), International Conference on Harmonization Good Clinical Practice, and local and national regulations. Written informed consent was provided by all patients prior to any study-related procedures.

The trial was designed, implemented, and overseen by the Steering Committee together with representatives of the sponsor, Vifor Pharma Ltd., Glattbrugg, Switzerland. ClinStar (Moscow, Russia) was responsible for on-site monitoring of sites in Russia and Ukraine. ICON (Dublin, Ireland) was responsible for on-site monitoring in other countries, in addition to data collection, data management, and data analysis. All analyses were performed according to a pre-defined statistical analysis plan validated by the sponsor Vifor Pharma Ltd. An independent Data Safety Monitoring Board, with no direct contact with the study site personnel nor with patients, reviewed safety data on an ongoing basis. The independent Clinical Endpoint Committee adjudicated all hospitalizations and deaths. The manuscript was prepared and submitted for publication by the Steering Committee. The authors had access to the study data and vouch for the accuracy and completeness of the reported analyses. The trial is registered at ClinicalTrials.gov, NCT01453608.

### Participants

Eligible patients included stable ambulatory HF patients in New York Heart Association (NYHA) class II or III, with left ventricular ejection fraction (LVEF) ≤45%, elevated natriuretic peptides (brain natriuretic peptide >100 pg/mL and/or N-terminal-pro-brain natriuretic peptide >400 pg/mL), presence of ID [defined as serum ferritin level <100 ng/mL, or between 100 and 300 ng/mL if transferrin saturation (TSAT) <20%] and haemoglobin (Hb) <15 g/dL (all at the screening visit). There was no lower limit for Hb, but subjects with an immediate need for transfusion were excluded. All subjects must have been capable of completing the 6 min walk test (6MWT). Patients with uncontrolled hypertension, infection, clinical evidence of current malignancy, or significantly impaired liver or renal function were excluded. There was no upper age limit. All detailed inclusion/exclusion criteria are presented in the design paper^17^ and in the protocol (see Supplementary material online).

### Randomization

At the baseline visit, prior to commencing treatment, clinical history, physical examination, 12-lead electrocardiogram, assessment of NYHA class, 6MWT, and health-related quality of life were obtained for each patient. Randomization was achieved using a central interactive voice response system to allocate patients to treatment groups and avoid selection bias. Eligible patients were randomly assigned in a 1 : 1 ratio to receive either i.v. iron or placebo (normal saline). Subjects were stratified by site and by Hb levels (two strata: subjects with Hb <12.0 g/dL vs. Hb ≥12.0 g/dL), stratification was incorporated into this study to help ensure a balance of baseline Hb across treatment groups.

### Therapy and blinding

Intravenous iron was given as ferric carboxymaltose solution [Ferinject^®^/Injectafer^®^ Vifor Pharma (FCM)]. Study medication was given as undiluted bolus i.v. injections of 10 or 20 mL (which is the amount of FCM that is equivalent to 500 or 1000 mg of iron, respectively) administered over at least 1 min. Normal saline [0.9% weight/volume (w/v) NaCl] was administered as placebo as per the instructions for active therapy.

Study drug (FCM or placebo) was administered in doses based on subject weight and Hb value at screening, according to the scheduled dosing scheme (see the design paper^17^ and the protocol in the Supplementary material online, Appendix for details). This included both therapy dosing (correction phase) and maintenance dosing (maintenance phase). In summary, total FCM doses were between 500 and 2000 mg iron FCM (or equivalent volume of placebo solution) in the therapy phase (dosed at baseline and Week 6), and thereafter maintenance FCM dosing of 500 mg iron (or equivalent volume of placebo solution) at each of Weeks 12, 24, and 36, if ID was still present (criteria for ID were re-assessed at each visit).

Each administration of study drug occurred after completion of all applicable study-related assessments (including quality of life assessments and collection of blood samples).

Ferric carboxymaltose is a dark brown and cannot easily be masked from placebo (0.9% saline). Therefore, unblinded study personnel (at least one physician) not involved in any study assessments for efficacy or safety were responsible for preparing and administering the study treatment injections in black syringes and using a curtain (or similar) to maintain subject blinding. The central laboratory results on iron metabolism markers and Hb were sent only to the unblinded study personnel who were responsible for evaluating these parameters for subsequent dosing and/or other intervention, if applicable.

### Study end-points

The primary end-point for the study was the change in 6MWT distance from baseline to Week 24.

The details of 6MWT are presented in the protocol (see Supplementary material online) and in the design paper.^17^ In summary, subjects were advised to take only a light meal and not to have undertaken vigorous exercise within 2 h prior to the test. The 6MWTs were planned to take place shortly after breakfast (i.e. early morning) or lunch (i.e. early afternoon). All tests were performed along a flat, straight corridor with a hard surface, at least 25 m long with turnaround points marked by two chairs at each end of the measured course. Prior to testing, vital signs were measured in a sitting position after a rest of 10 min and subjects completed the Fatigue Score (assessed using a 10-point visual analogue scale, ranging from 1 for no fatigue to 10 for very severe fatigue). Subjects were instructed to walk the length of the course at their own pace while attempting to cover as much ground as possible in 6 min. The person supervising the 6MWT encouraged the subject verbally at frequent intervals. Subjects were allowed to rest on the chairs during the test, but were encouraged to resume walking as soon as they felt physically able to do so. The distance walked in 6 min, to the nearest meter, was recorded. Every effort was made to have the same member of the site supervising all 6MWTs for a specific subject.

Secondary end-points included changes in NYHA class, Patient Global Assessment (PGA), 6MWT distance, Fatigue Score and health-related quality of life [evaluated using Kansas City Cardiomyopathy Questionnaire (KCCQ), European Quality of Life 5D (EQ-5D) questionnaire] assessed at Weeks 6, 12, 24, 36, and 52. Additionally, the following secondary outcome-related end-points were assessed, in which deaths were censored in the analysis of HF hospitalizations:^17^

- rate of any hospitalization, rate of hospitalization for any cardiovascular reason, and rate of hospitalization due to worsening HF;
- time to first hospitalization for any reason, time to first hospitalization for any cardiovascular reason and time to first hospitalization due to worsening HF;
- time to death for any reason, time to death for any cardiovascular reason, and time to death due to worsening HF.

Standardized definitions for cause of death or hospitalization were developed by members of the independent Clinical Endpoint Committee of CONFIRM-HF (for details see the design paper^17^. All such events were recorded throughout the study and adjudicated by the Clinical Endpoint Committee.

Additional secondary end-points were changes from baseline to Weeks 6, 12, 24, 36, and 52 in clinical laboratory panels (haematology, clinical chemistry, iron status, and cardiac biomarkers). Safety analysis included serious and non-serious adverse events, assessed up to Week 52.

### Statistical analysis

The sample size calculation for CONFIRM-HF was based on the expected change of 6MWT distance at Week 24, using the data reported in the FAIR-HF study.^18^ The mean difference between groups in the change of 6MWT distance from baseline at Week 24 in FAIR-HF was 29 m with a standard deviation (SD) of 72 m. Based on these assumptions, a sample size of 130 subjects per group (i.e. 260 patients in total) were required to detect a mean treatment effect of at least 29 m at Week 24 with 90% power using an alpha of 0.05 (two-sided). The sample size was increased to 150 subjects per group (300 total) to allow for some loss of information due to early study discontinuation.

The primary efficacy analysis was performed according to the intention-to-treat principle (ITT) on the full-analysis set (FAS), including all subjects who were randomized and in whom investigational drug treatment was started, and with efficacy data returned. Subjects were analysed according to the treatment group to which they were randomly assigned, i.e. irrespective of actual treatment received. In addition supportive analysis will be performed on the per-protocol analysis set.

The primary efficacy end-point, change in 6MWT from baseline to Week 24, included imputations for missing values from subjects who were hospitalized at that time or had died. For hospitalized patients, the worst non-null 6MWT result collected across the study was then used for the analysis. For subjects who died, a value of 0 was imputed. The primary efficacy analysis was conducted using an analysis of the covariance (ANCOVA) model on the change in 6MWT from baseline to Week 24, with adjustment for baseline 6MWT distance, Hb level at screening and country (Russia, Ukraine, Poland, and other European countries) on the FAS.

Supportive analyses were performed using the per-protocol set (those subjects included in the FAS without major protocol violations) and observed cases (without imputation for subjects that were hospitalized at the time or had died).

NYHA class missing values due to subjects who died were imputed using the worst possible assessment of class V, and subjects hospitalized during the planned assessment were attributed a value of class IV. Missing PGA values due to death were imputed as ‘died’ and missing PGA values due to hospitalization were imputed as ‘much worse’.

ANCOVA repeated measure models were used for the analysis of the continuous secondary end-points variables, and repeated measures polytomous regression for the non-continuous variables analysis. Time-to-event analyses were conducted using Kaplan–Meier estimators and log-rank tests. Hazard ratios (HRs) and corresponding 95% confidence intervals (CIs) were obtained from the proportional hazard ratio models. *Post hoc* sensitivity analyses included time-to-event analyses on composite end-points for death and any first hospitalization, death and first hospitalization for any cardiovascular reason or death and first hospitalization due to worsening HF. Further *post hoc* sensitivity analyses were conducted on the secondary end-point of hospitalization events due to worsening HF using negative binomial regression models where incidence rate ratios between treatment groups the 95% CIs and *P*-values were calculated.^19,20^

Safety analyses by summary statistics were performed on all subjects who received at least one dose of investigational drug or placebo. Follow-up for collection of key safety information was until 30 days after the end of the study, i.e. longer than for the efficacy information. Subjects were analysed according to the treatment they actually received.

## Results

**Figure 1:**
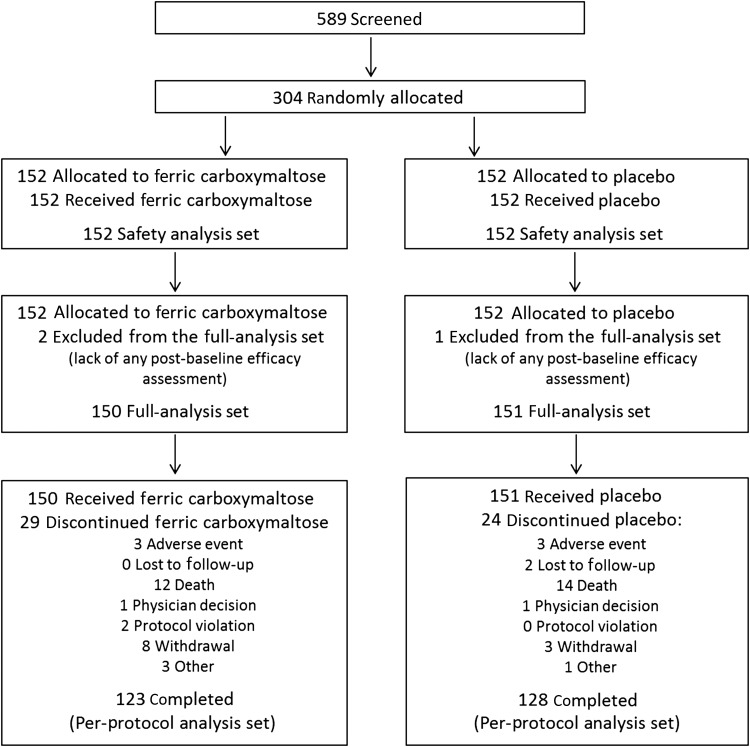
Enrolment and outcomes. The full analysis set comprised all patients who received at least one dose of randomized treatment and attended at least one post-baseline visit. The safety population included all patients who received at least one dose of randomized treatment. Supportive analyses were performed using the per-protocol set (those subjects who participated in the trial included in the full-analysis set without major protocol violations).

In total, 304 patients were enrolled (FCM, 152; placebo, 152) at 41 sites across 9 countries (Austria, Italy, Poland, Portugal, Russia, Spain, Sweden, UK, Ukraine). All randomized patients received at least one dose of study treatment (*Figure 1*). Three out of the 304 randomized patients were excluded from the FAS for efficacy analyses due to lack of any post-baseline efficacy assessment, but had continued in the study (note: none of these three patients had a hospitalization or death event). The clinical characteristics of these 301 patients are presented in *Table 1*. Baseline clinical and laboratory characteristics and the use of various cardiac medications at the time of enrolment were similar between the two treatment groups.

**Table 1:** Baseline Demographic and Clinical Characteristics (FAS)

Variable	FCM	Placebo
	(N = 150)	(N = 151)
	Mean (SD)	Mean (SD)
Age *yrs*	68.8 (9.5)	69.5 (9.3)
Female sex *n (%)*	67 ( 45)	74 ( 49)
White race *n (%)*	149 ( 99)	150 ( 99)
NYHA class *n (%)*		
II	80 ( 53)	91 ( 60)
III	70 ( 47)	60 ( 40)
LVEF *%*	37.1 (7.5)	36.5 (7.3)
Body Mass *kg*	78.6 (14.0)	80.8 (18.4)
Body Mass Index *kg/m*^*2*^	28.3 (4.6)	29.1 (5.7)
Blood pressure *mm Hg*		
Systolic	125 (14)	124 (13)
Diastolic	75 (8)	75 (8)
Pulse *beats/minute*	69 (11)	71 (11)
6-Minute walk test distance *m*	288 (98)	302 (97)
Ischemic cause of heart failure *n (%)*	125 ( 83)	126 ( 83)
Quality of Life Assessments		
Fatigue score	5.5 (1.6)	5.3 (1.7)
KCCQ score	59.0 (17.3)	58.8 (17.9)
EQ-5D VAS	54.7 (15.0)	54.1 (16.3)
Cardiovascular Risk Factor *n (%)*		
Hypertension	130 ( 87)	130 ( 86)
Dyslipidaemia	98 ( 65)	98 ( 65)
Diabetes mellitus	38 ( 25)	45 ( 30)
Smoking	54 (36)	41 (27)
Medical History *n (%)*		
Atrial fibrillation	66 ( 44)	73 ( 48)
Myocardial infarction	90 ( 60)	90 ( 60)
Angina pectoris	98 ( 65)	91 ( 60)
Stroke	21 ( 14)	24 ( 16)
Coronary revascularization	46 ( 31)	39 ( 26)
Laboratory Measurements		
Hb *g/dL*	12.37 (1.41)	12.42 (1.30)
Ferritin *ng/mL*	57.0 (48.4)	57.1 (41.6)
< 100 ng/ml *n (%)*	136 (91)	133 (88)
TSAT *%*	20.2 (17.6)	18.2 (8.1)
CRP *mg/L*	5.19 (9.00)	6.00 (11.60)
BNP *pg/mL*	772 (995)	770 (955)
NT Pro-BNP *pg/mL*	2511 (5006)	2600 (4555)
Sodium *mmol/L*	143 (3)	142 (5)
Potassium *mmol/L*	4.69 (0.54)	4.63 (0.55)
ALT *U/L*	21.1 (18.9)	18.7 (9.9)
AST *U/L*	26.2 (19.6)	23.5 (8.6)
eGFR *mL/min/1.73m*^*2*^	66.4 (21.7)	63.5 (20.9)
Concomitant treatment *n (%)*		
Diuretic	132 (88)	139 (92)
ACE inhibitor	116 (77)	118 (78)
ARB	34 (23)	37 (25)
Digitalis glycoside	29 (19)	40 (27)
Beta-blocker	133 (89)	139 (92)
Antithrombotic agents	142 (95)	144 (95)
Lipid-lowering therapy	105 (70)	110 (73)
Insulin and analogues	18 (12)	20 (13)
Oral hypoglycaemic agent	26 (17)	32 (21)

### Follow-up

Of the 150 patients assigned to FCM, 29 (19.3%) patients did not complete the study of whom 12 (8.0%) patients died. Of the 151 patients assigned to placebo, 24 (15.9%) patients did not complete the study of whom 14 (9.3%) patients died. Two patients from the placebo group were lost to follow-up (*Figure 1*). One patient from the FCM group died within the 30 day safety follow-up period after completing the study.

### Primary end-point

Baseline values [mean (SD)] of 6MWT distance were similar between treatment groups [288 (98) m vs. 302 (97) m, respectively, FCM vs. placebo]. At Week 24, in the FCM an increase in 6MWT distance by 18 ± 8 m was detected, whereas in the placebo group there was a decrease in 6MWT distance by 16 ± 8 m (both least squares mean ± standard error). It resulted in a significant difference in changes in 6MWT distance at Week 24 in FCM vs. placebo of 33 ± 11 m (least squares mean ± standard error) , *P* = 0.002.

### Secondary end-points

**Figure 2:**
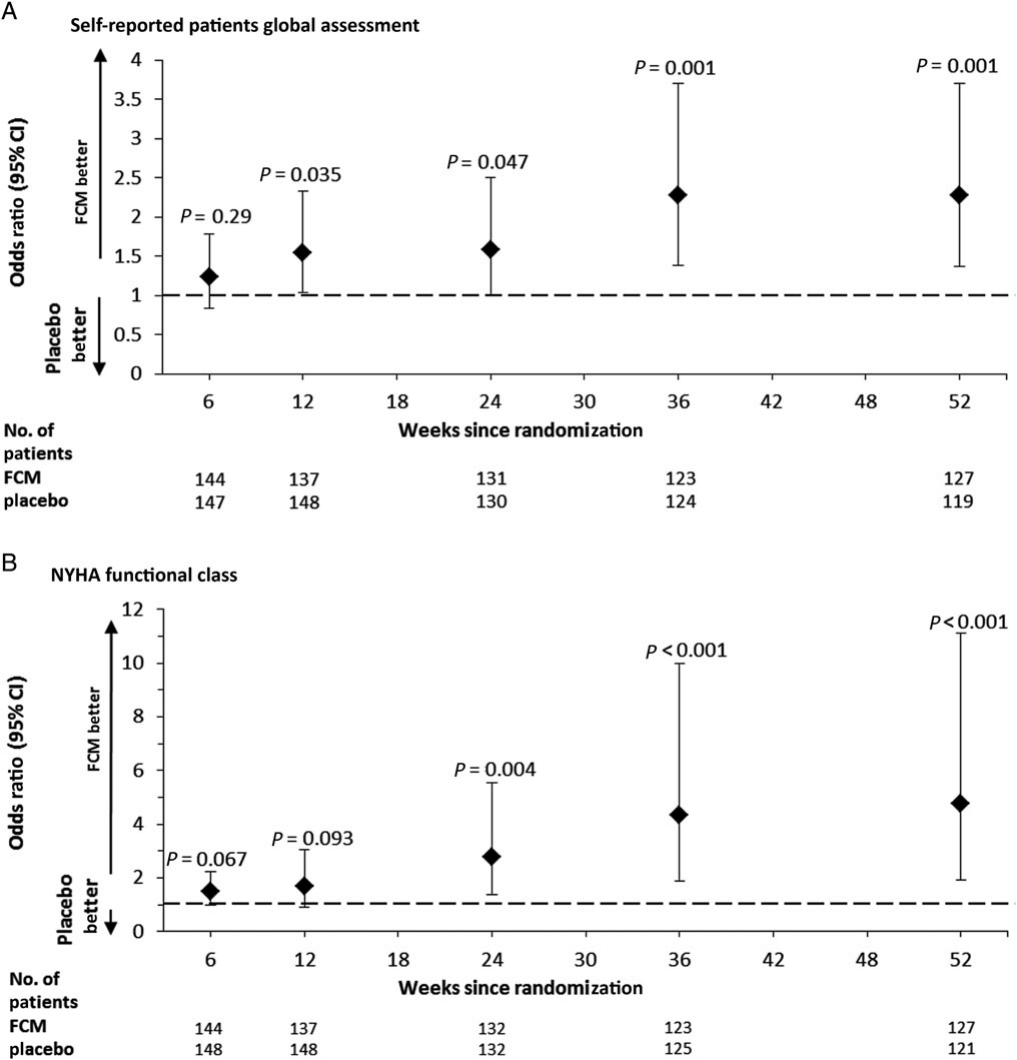
Patient Global Assessment and NYHA Functional Class over Time (full-analysis set). The data presented are odds ratios for patient global assessment (*A*) and NYHA functional class (*B*) for the ferric carboxymaltose group when compared with the placebo, of being in a better category of patient global assessment (*A*) and NYHA functional class (*B*). In those panels, the *P*-values are for the comparison between the two study groups, and the I bars denote the 95% confidence intervals.

**Figure 3:**
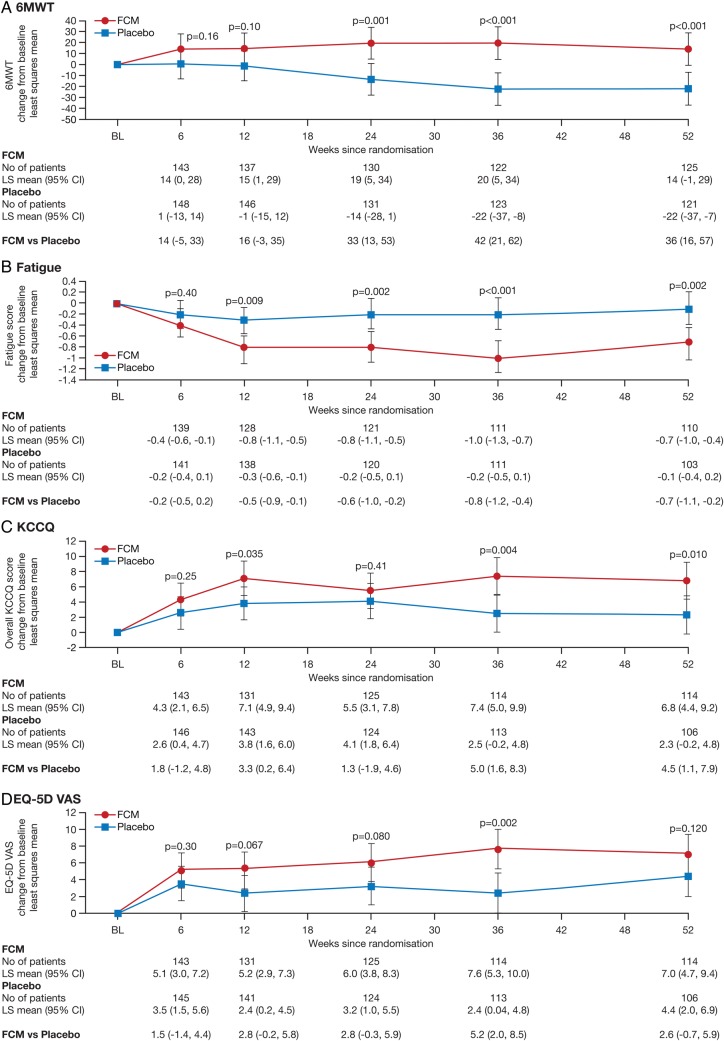
6-Min Walk Test, Fatigue and Quality of Life Score over Time (full-analysis set). Shown are data across the study period using a repeat measures model for the changes (least squares mean with the 95% confidence intervals on the 6-min walk test (*A*), Fatigue score (assessed using a 10-point visual analogue fatigue scale, ranging from 1 for no fatigue to 10 for very severe fatigue) shown in (*B*), Kansas City Cardiomyopathy questionnaire (KCCQ) score (on which the overall score ranges from 0 to 100, with a higher score indicating a better QoL) shown in (*C*), and the European Quality of Life-5 Dimensions (EQ-5D) Visual Analogue Scale (on which the score ranges from 0 to 100, with higher scores indicating better health) shown in (*D*).

The use of FCM, when compared with placebo, showed a significant benefit in PGA from Week 12 onwards (*P* = 0.035 at Week 12, *P* = 0.047 at Week 24, and *P* = 0.001 at Weeks 36 and 52) and NYHA class from Week 24 onwards (*P* = 0.004 at Week 24 and *P* < 0.001 at Weeks 36 and 52) (*Figure 2A* and *B*). By using a repeated measures, model significant improvements were also seen in the differences in changes in 6MWT distance at Weeks 36 (42 m with 95% CI of 21–62, *p* < 0.001) and Weeks 52 (36 m with 95% CI of 16–57, *p* < 0.001) (*Figure 3A*). Significant reductions in the Fatigue Score were observed from Week 12 onwards (*P* = 0.009 at Week 12, *P* = 0.002 at Week 24, and Week 52, *P* < 0.001 at Week 36) for the FCM group compared with placebo (*Figure 3B*). A beneficial effect on QoL, as evaluated by the overall KCCQ score, was observed in the FCM group at Weeks 12, 36, and 52 (*P* < 0.05 for all comparisons) (*Figure 3C*). The EQ-5D health state score showed a benefit for FCM over placebo throughout the study but achieved significance only at Week 36 (*P* = 0.002) (*Figure 3D*).

**Figure 4:**
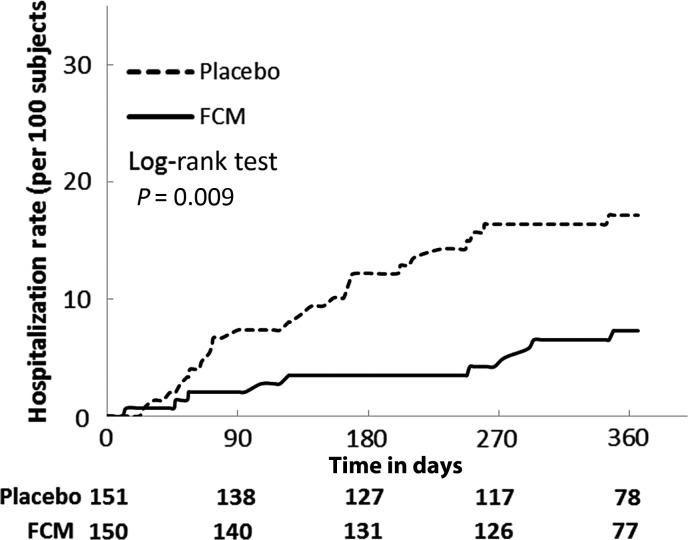
Time to first hospitalization due to worsening heart failure. The time to first hospitalization due to worsening heart failure was estimated using the Kaplan–Meier method, on the full-analysis set. Subjects were censored at their death, study completion, or withdrawal date.

During the study, 76 patients were hospitalized at least once [FCM: 32 (21%), placebo: 44 (29%) patients]. The time-to-event analysis indicated an HR of 0.71 with a 95% CI of (0.45–1.12) (*P* = 0.14) (*Table 2*). There were 46 hospitalizations for any reason in the FCM group and 69 in the placebo group. Treatment with FCM was associated with a significant reduction in the risk of hospitalization due to worsening HF with a time-to-event analysis returning an HR of 0.39 with a 95% CI of (0.19–0.82) (*P* = 0.009) (*Table 2*, *Figure 4*). The incidence of all-cause death was similar in both groups (FCM: 8.9, placebo: 9.9 per 100 patient-year at risk) (*Table 2*).

**Table 2:** Hospitalizations and deaths (full-analysis set)

End-point or event	FCM (*n* = 150)		Placebo (*n* = 151)			
	Total number of events	Incidence/100 patient-years at risk	Total number of events	Incidence/100 patient- years at risk	Time to first event hazard ratio 95% CI	*P*-value
Death	12	12 (8.9)	14	14 (9.9)	0.89 (0.41– 1.93)	0.77
Death for any cardiovascular reason	11	11 (8.1)	12	12 (8.5)	0.96 (0.42– 2.16)	0.91
Death due to worsening HF	4	4 (3.0)	3	3 (2.1)	1.39 (0.31–6.21)	0.67
Death due to other cardiovascular reason	7	7 (5.2)	9	9 (6.4)	0.81 (0.30–2.17)	0.68
Hospitalizations	46	32 (26.3)	69	44 (37.0)	0.71 (0.45–1.12)	0.14
Hospitalizations for any cardiovascular reason	26	21 (16.6)	51	33 (26.3)	0.63 (0.37–1.09)	0.097
Hospitalizations due to worsening HF	10	10 (7.6)	32	25 (19.4)	0.39 (0.19–0.82)	0.009
Hospitalizations due to other cardiovascular reason	16	13 (10.0)	19	15 (11.0)	0.91 (0.43–1.92)	0.81
*Post hoc* Analyses						
Hospitalizations or death	58	38 (31.2)	83	50 (42.1)	0.75 (0.49–1.14)	0.17
Hospitalizations for any cardiovascular reason or death	38	28 (22.1)	65	40 (31.9)	0.70 (0.43–1.13)	0.14
Hospitalizations due to worsening HF or death	22	18 (13.7)	46	33 (25.6)	0.53 (0.30–0.95)	0.03
Hospitalizations due to other cardiovascular reason or death	28	23 (17.7)	33	25 (18.3)	0.97 (0.55–1.70)	0.91
Hospitalizations or death for any cardiovascular reason	37	27 (21.3)	63	38 (30.3)	0.71 (0.43–1.16)	0.16

Incidence/100 patient-years at risk are computed using the number of subjects with the end-point/event adjusted on the total length of exposure while the subjects are still at risk [before observing the first event or before completing the study for subjects without any event (censored)].

In *post hoc* sensitivity analysis, we found that the combined risk of first hospitalization due to worsening HF or all-cause death was significantly lower in the FCM group [HR (95% CI): 0.53 (0.30–0.95), *P* = 0.03] (*Table 2*). The *post hoc* sensitivity analysis of recurrent events on the number of hospitalizations due to worsening HF using the negative binomial regression models confirmed positive treatment effect of FCM with an incidence rate ratio (95% CI) of 0.30 (0.14–0.64), *P* = 0.0019 compared with placebo (in total 10 hospitalizations due to worsening of HF in the FCM and 32 in the placebo group during the study).

Laboratory values for serum ferritin, TSAT, and Hb showed an increase in the FCM group at Weeks 24 and 52 and were significantly different between the two treatment groups (all *P* < 0.001). Overall, the mean treatment effect on ferritin and TSAT (adjusted for baseline) in patients assigned to FCM compared with placebo was 265 ± 19 ng/mL and 8.9 ± 1.1% at Week 24, and 200 ± 19 ng/mL and 5.7 ± 1.2% at Week 52 (all *P* < 0.001). The corresponding mean differences for Hb (adjusted for baseline) were 0.6 ± 0.2 and 1.0 ± 0.2 g/dL, at Weeks 24 and 52, respectively (all *P* < 0.001).

### Subgroup analyses

**Figure 5:**
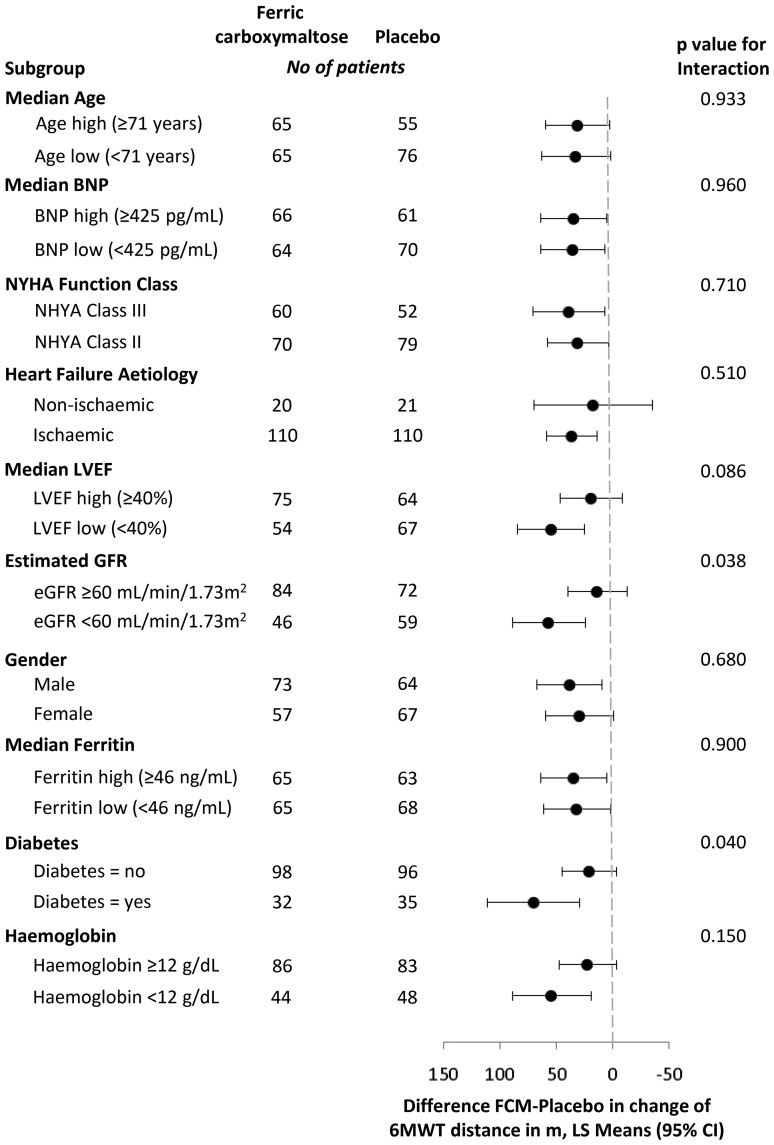
Subgroup analyses for 6-Min Walk Test. Shown are data of 6MWT distance change from baseline to Week 24 analysis results, using ANCOVA analyses with each subgroup as a covariate, and treatment and the interaction between treatment and subgroup as covariates. The least squares mean of the difference between ferric carboxymaltose and placebo groups and the 95% confidence intervals are displayed for each subgroup. The *P*-values of the interaction term (treatment and subgroups) are presented.

In all subgroups examined, the treatment effect was preserved. A consistent improvement in 6MWT distance at Week 24 in patients treated with FCM when compared with placebo was demonstrated. For the majority of subgroups, there was no significant interaction (*Figure 5*). Where the interaction for the subgroups was statistically significant, i.e. in those with/without diabetes mellitus (*P* = 0.04) and impaired/preserved renal function (*P* = 0.038), the magnitude of the benefit for FCM over placebo varies, but it is not indicative of a different direction of effect in any of these subgroups. The original, pre-specified subgroup for baseline ferritin examined those subjects with baseline ferritin levels at <100 or ≥100 ng/mL. However, the number of subjects in the latter group was very small (14 in the FCM group and 18 in the placebo group) and made the results of this analysis uninterpretable. Therefore, we examined subjects with baseline ferritin above and below the median ferritin value as a *post hoc* analysis (*Figure 5*).

In the primary efficacy analysis adjustment for country (Russia, Ukraine, Poland considered separately, and other European countries pooled together) revealed no significant interaction (*P* = 0.30), which indicates that treatment effect on 6MWT at Week 24 is consistent without major regional outliers.

### Safety analyses

The overall incidence of investigator reported adverse events, serious adverse events and adverse events leading to study discontinuation were similar in both groups (*Table 3*).

**Table 3:** List of adverse events (safety amalysis set)

Safety end-point or event	FCM (*n* = 152) *n* (%) events	Placebo (*n* = 152) *n* (%) events
Subjects with at least one AE	121 (79.6) 555	115 (75.7) 547
Subject with at least one severe AE	21 (13.8) 31	27 (17.8) 54
Subject with at least one serious AE	43 (28.3) 68	53 (34.9) 106
Subject with at least one AE leading to study drug withdrawal	14 (9.2) 14	19 (12.5) 19
Subject with at least one severe drug-related AE	0 (0.0) 0	0 (0.0) 0
Subject with at least one serious drug-related AE	0 (0.0) 0	0 (0.0) 0
Subject with at least one drug-related leading to study drug withdrawal	1 (0.7) 1	0 (0.0) 0
Subject with at least one drug-related AE	14 (9.2) 24	5 (3.3) 7
General disorders and administration site conditions	9 (5.9) 9	2 (1.3) 2
Skin and subcutaneous tissue disorders	4 (2.6) 4	0 (0.0) 0
Nervous system disorders	2 (1.3) 3	1 (0.7) 1
Gastro-intestinal disorders	2 (1.3) 3	0 (0.0) 0
Vascular disorders	1 (0.7) 2	1 (0.7) 1
Investigations	1 (0.7) 1	2 (1.3) 2
Ear and labyrinth disorders	1 (0.7) 1	0 (0.0) 0
Injury, poisoning and procedural complications	1 (0.7) 1	0 (0.0) 0
Cardiac disorders	0 (0.0) 0	1 (0.7) 1

*n*, number of subjects experiencing at least one time the considered event; %, percentage of above in the total number of subjects in the group; events, total number of events.

No severe allergic reactions related to the study treatment were reported. Of the patients reporting treatment-related adverse events with FCM, two patients experienced injection site discolouration, four patients reported feeling hot, and skin discolouration, urticaria, rash, and erythema were each reported by one patient.

No differences were observed between the two treatment groups with respect to investigator reported adverse events related to laboratory test results.

### Dosing

In the FCM arm, the mean and median total dose was 1500 mg of iron during the 1-year study period, with a dosing range of 500–3500 mg of iron. Over 75% of the patients required a maximum of two injections of FCM to correct and maintain the iron parameters.

## Discussion

The CONFIRM-HF study shows that the treatment of stable, symptomatic, iron-deficient HF patients with i.v. iron (FCM) results in sustainable improvement in functional capacity as measured over a 1-year period using the 6-MWT walking test. These favourable results were consistent across all pre-specified subgroups including patients with and without anaemia. The beneficial effects of treatment with FCM were confirmed by concomitant improvement in patients functional status and quality of life throughout the study. Importantly, patients treated with FCM demonstrated significantly reduced risk of hospital admission due to worsening HF during 1-year follow-up. Long-term correction of ID with FCM was supported by a good safety profile and was well-tolerated.

Current guidelines of the European Society of Cardiology for the management of HF recognize ID as a common and clinically relevant comorbidity, complicating the natural course of the disease, and recommend to actively search for this abnormality using laboratory parameters in all patients with HF.^2^ At the same time, there is a relatively weaker recommendation to manage ID in such patients, which mainly results from a paucity of evidence-based data confirming the benefits of iron therapy.^2,14^ In fact, there is only one medium-size, double-blind, placebo-controlled clinical trial (FAIR-HF) showing beneficial effects of i.v. iron therapy with FCM on functional status, exercise intolerance and QoL in ID patients with HF over a 6-month follow-up period.^18^ There is no doubt that for a stronger recommendation of ID as a valid therapeutic target in HF, additional well-designed and controlled studies with longer follow-up period are needed. To this end, CONFIRM-HF was designed and executed.

Noteworthy, the results of CONFIRM-HF should be viewed as adding incremental and clinically relevant information over already existing data in the following aspects:

- selection of a different and—compared with FAIR-HF—more objective primary end-point (changes in 6MWT distance) in order to use more robust method for the assessment of the clinical status of HF patients;
- documenting longer-term sustainability of beneficial effects of treatment with FCM with acceptable safety profile (i.e. for 12 months compared with 6 months or less in prior studies);
- providing intriguing data on significant risk reduction of the hospitalization due to HF worsening;
- offering a simplified and more clinically applicable method of ID therapy with FCM.

All these aspects will be briefly discussed below.

In FAIR-HF, the primary end-point was based on the evaluation of NYHA class and PGA.^18^ In CONFIRM-HF, we chose changes in 6MWT distance as the primary end-point, because it is a more robust method assessing the clinical status of HF patients.^21,22^ The 6MWT is a well-established, reproducible method for the assessment of functional capacity, sensitive to changes in self-reported symptoms,^22^ which has been previously used in numerous HF studies evaluating the effects of different interventions.^23^ We expected, that by targeting ID, which impedes oxygen transportation and/or utilization we would be able to improve patients' exercise tolerance.^15^ Our results demonstrate indeed, that therapy with FCM significantly improved patients' functional capacity. Beneficial effects of FCM were already seen at Weeks 6 and 12 (strong trend favouring FCM), reaching statistical significance at Week 24 and were sustained until the end of 1-year follow-up. The magnitude of the treatment effect of FCM on the 6MWT distance, exceeding 30 m in the last 6-month study period, is robust and clinically meaningful. In previous interventional studies, such beneficial effects have only been seen with cardiac resynchronization therapy.^23^ Importantly, improvement in 6MWT distance was seen across all examined subgroups, including patients with and without anaemia, which further challenges the traditional view linking adverse consequences of ID with anaemia. Cardiopulmonary exercise testing with the assessment of peak oxygen consumption is another method applied to evaluate functional capacity in HF, which can provide even more objective information on exercise tolerance than 6MWT. Currently, there is an ongoing clinical trial using this method (www.clinicaltrial.gov: EFFECT-HF, NCT01394562), which will provide complementary information on the effects i.v. iron therapy with FCM in iron-deficient HF patients.

Patients included in this study represent a contemporary population of stable, systolic HF with optimized medical management. Of importance, compared with the FAIR-HF study, we recruited nearly equal numbers of patients in NYHA class II and III (compared with 18% NYHA class II patients in FAIR-HF) with higher LVEF (mean LVEF—37 vs. 32% in FAIR-HF). The benefit was seen regardless of clinical severity (as evidenced by no interaction between NYHA class, LVEF and BNP level with treatment effect), which further broadens the clinical applicability of our results.

In the subgroup analyses, the presence of diabetes and impaired renal function interacted with treatment effects. However, these interactions are not for the direction of effect but only for its magnitude (with a greater benefit for sicker patients with diabetes and impaired renal function). Interestingly, these findings may have important clinical implications allowing us to identify HF patients who could potentially benefit most from i.v. iron therapy. Of note, it has recently been demonstrated that ID is common in patients with coronary artery disease with concomitant type 2 diabetes mellitus and independently predicts poor outcome.^9^ Heart failure patients with renal dysfunction are prone to develop ID, which often coincides with low-Hb level.^24^ Therapy with i.v. iron is able to correct these ominous abnormalities. Interestingly, Toblli *et al*.^25^ demonstrated that in anaemic HF patients with ID and renal dysfunction, short-term i.v. iron therapy resulted in significant improvement in renal function. At this stage, however, these intriguing findings need to be considered as hypothesis-generating only, and further tested in prospective clinical trials.

In this study, beneficial effects of ID correction with FCM were further confirmed by observed improvement in the other indices of functional capacity (NYHA class, PGA, Fatigue score) as well as in QoL throughout the whole study period. This is particularly important, as despite modern improvement in HF management, a considerable proportion of patients remains symptomatic. Hence, well-tolerated therapies with good safety profiles, improving symptoms in the long-term perspective are eagerly awaited.

In evaluating the effects of new therapies in the settings of chronic HF one would expect these would improve patients' clinical status and QoL, reduce the risk of deterioration (i.e. hospital admission due to worsening HF), and finally prolong survival. Recently, investigators observed a striking reduction in mortality in patients with chronic HF with a parallel increase in the hospital admission rate due to worsening HF.^26^ As hospitalizations due to worsening HF are always related to poor outcome and impairment of patient's quality of life and constitute an economic burden for society,^27^ there is evident need for their prevention. In this context, the results of CONFIRM-HF showing that FCM treatment was related to a significant risk reduction in first hospital admissions due to worsening HF are of particular interest. Additional analysis taking into account all recurrent HF hospitalizations showed an even stronger effect with a rate ratio (FCM vs. placebo) of 0.30. Among recently introduced pharmacological therapies, only ivabradine^28^ has demonstrated such results. Although there was no difference in the numbers of deaths between groups, a 1-year follow-up may not be adequate to detect any mortality difference. We are aware that the study was not designed primarily to address the morbidity/mortality aspect of ID therapy with FCM, but our results constitute a strong background for such a study to be performed in the near future.

As in previous studies,^16,18^ our patient population was identified on the basis of laboratory biomarkers of ID—ferritin and TSAT. However, in contrast to these studies we used a simplified dosing regimen for FCM recently proposed by Evstatiev *et al*.^29^ based on weight and Hb levels. These authors demonstrated that this simplified dosing regimen is superior in efficacy to dosing using the traditional Ganzoni formula^30^ with an accompanying good safety profile. In the FCM arm, the median total dose was 1500 mg of iron during the 1-year study period (with a dosing range of 500–3500 mg iron) and over 75% of the patients required a maximum of two injections of FCM to correct ID and maintain the iron parameters within the normal range. Additionally, we extended the previous experience with FCM therapy in non-anaemic ID patients which included patients with Hb levels between 9.5 and 13.5 g/dL,^18^ whereas in this study only patients with Hb values >15 g/dL were excluded.

In most of the recent studies, ID in HF patients has been corrected using i.v. iron with favourable results. Thus, a relevant question arises, whether similar results would be observed with oral iron therapy. This complex problem remains as yet poorly investigated and unanswered. There are several premises favouring i.v. iron and practicality seems to be the most obvious one. To replete ID in HF, which is typically estimated in a range exceeding 1000 mg, several months of oral therapy would be required with subsequent risk of poor tolerance. In contrast, with FCM as studied in FAIR-HF and CONFIRM-HF, a low risk of adverse effects is observed and only few injections are needed to treat ID (in CONFIRM-HF over 75% of the patients required a maximum of two injections of FCM to correct and maintain iron therapy). Recent experimental evidence demonstrates disrupted regulatory mechanisms of duodenal iron transportation systems in animals with induced HF and ID.^31^ On the other hand, iron absorption has never been investigated in HF patients, and whether the phenomena described in rodents would play any role in a clinical setting of HF is entirely unknown. It is also tempting to link ID in HF with inflammation, and to hypothesize about the leading role of elevated hepcidin, which also blocks iron absorption. Recent studies, however, report rather low-hepcidin levels in HF patients and no association between pro-inflammatory activation (as evidenced by circulating IL-6) and hepcidin levels.^13,32,33^ There is only one small clinical study showing the advantage of i.v. iron therapy over oral iron on exercise capacity in anaemic iron-deficient HF patients,^34^ but with only 18 subjects analysed it is far from being conclusive. Therefore, there is a need to evaluate the efficacy of oral iron therapy in iron-deficient HF patients in adequately large, prospective, randomized clinical trial. To our knowledge such a trial is planned (www.clinicaltrial.gov: IRONOUT, NCT02188784).

In conclusion, treatment of stable, symptomatic, iron-deficient HF patients with ferric carboxymaltose over a 1-year period results in sustained improvement in functional capacity, symptoms and quality of life, and may reduce hospitalizations due to worsening HF.

## Supplementary material

Supplementary material is available at *European Heart Journal* online.

## Funding

This work was supported by Vifor Pharma Ltd., Glattbrugg, Switzerland.

**Conflict of interest:** P.P. received honoraria from Vifor Pharma Ltd. as member of the CONFIRM-HF Steering Committee, is a consultant and has received honoraria for speaking from Vifor Pharma Ltd. and Amgen, Inc. P.P. also reports having received research grants from Vifor Pharma Ltd.; D.V. received honoraria as member of the CONFIRM-HF Steering Committee from Vifor Pharma Ltd. and board membership fees from Vifor Pharma Ltd. and Amgen, Inc.; T.M. received honoraria as member of the CONFIRM-HF Steering Committee and board membership fees from Vifor Pharma Ltd.; G.F. and F.R. received board membership fees from Vifor Pharma Ltd. G.F. and F.R. received honoraria as members of the CONFIRM-HF Clinical Endpoint Committee from Vifor Pharma Ltd.; J.C.C., V.M., L.T., G.E., M.K., and A.P. received honoraria from Vifor Pharma Ltd. as members of the CONFIRM-HF Steering Committee; C.M. is an employee of Vifor Pharma Ltd. owning stocks in Galenica.; V.L. and B.R. are employees of Vifor Pharma Ltd.; S.D.A. has received honoraria from Vifor Pharma Ltd. for consultancy, lectures, clinical trial committee work, and/or trial adjudication work. S.D.A. also has received research grants from Vifor Pharma Ltd.
